# A trade off between *mlo* resistance to powdery mildew and increased susceptibility of barley to a newly important disease, Ramularia leaf spot

**DOI:** 10.1093/jxb/ert452

**Published:** 2014-01-07

**Authors:** Graham R. D. McGrann, Anna Stavrinides, Joanne Russell, Margaret M. Corbitt, Allan Booth, Laetitia Chartrain, William T. B. Thomas, James K. M. Brown

**Affiliations:** ^1^Department of Crop Genetics, John Innes Centre, Norwich Research Park, Norwich, NR4 7UH, UK; ^2^The James Hutton Institute, Invergowrie, Dundee DD2 5DA, Scotland, UK

**Keywords:** Biotrophic pathogens, *Blumeria graminis*, disease resistance, *Hordeum vulgare*, necrotrophic pathogens, plant breeding, *Ramularia collo-cygni*, *ROR genes*.

## Abstract

Ramularia leaf spot (RLS), caused by the fungus *Ramularia collo-cygni*, is a serious, recently emerged disease of barley in Europe and other temperate regions. This study investigated the trade off between strong resistance to powdery mildew conferred by *mlo* mutant alleles and increased susceptibility to RLS. In field trials and seedling tests, the presence of *mlo* alleles increased severity of RLS. Genetic analysis of a doubled-haploid population identified one quantitative trait locus for susceptibility to RLS, colocalizing with the *mlo-11* allele for mildew resistance. The effect of *mlo-11* on RLS severity was environmentally sensitive. Analysis of near-isogenic lines of different *mlo* mutations in various genetic backgrounds confirmed that *mlo* alleles increased RLS severity in seedlings and adult plants. For *mlo* resistance to mildew to be fully effective, the genes *ROR1* and *ROR2* are required. RLS symptoms were significantly reduced on *mlo-5 ror* double mutants but fungal DNA levels remained as high as in *mlo-5* single mutants, implying that *ror* alleles modify the transition of the fungus from endophytism to necrotrophy. These results indicate that the widespread use of *mlo* resistance to control mildew may have inadvertently stimulated the emergence of RLS as a major disease of barley.

## Introduction

Breeders of crop species need to select plants with adequate resistance to all significant diseases in the region for which seed of new cultivars is to be sold. They must also combine disease resistance with other commercially important traits, such as yield (Summers and Brown, 2013). Durable disease resistance can contribute to stability of yield despite annual variation in biotic and abiotic stresses and thus promote economic and environmentally sustainable food production. If a gene increases resistance to an important disease but has an undesirable impact on other traits, understanding the trade offs between these positive and negative effects can give insights into the gene’s function and inform rational decisions about the likely value of the gene for plant breeding (Brown and Rant, 2013). Here, this work reports that *mlo* resistance to powdery mildew of barley, one of the most successful and widely used durable resistances to an important crop disease, has the detrimental effect of increasing susceptibility to another disease, Ramularia leaf spot, which has become important over the period that *mlo* has been widely deployed in cultivars.

A major success in plant breeding for disease resistance is the broad-spectrum, durable control of powdery mildew (*Blumeria graminis* f. sp. *hordei*) of barley conferred by recessive alleles of *Mlo*. Wild-type *Mlo* encodes a seven transmembrane-domain protein that interacts with calmodulin to negatively regulate plant defence and promote susceptibility to powdery mildew fungi in both monocot and dicot plants (Büschges *et al.*, 1997; Kim *et al.*, 2002; Consonni *et al.*, 2006). Loss-of-function mutations in *Mlo* result in broad-spectrum resistance to mildew by restricting fungal penetration (Piffanelli *et al.*, 2002). Mutations in *Mlo* have provided resistance against *B*. *graminis* f. sp. *hordei* for nearly 40 years (Jørgensen *et al.*, 1992). The most commonly used alleles in agriculture are *mlo-11*, from an Ethiopian landrace, which is now used in about half of all European spring barley cultivars, and, in a few cultivars, *mlo-9*, an artificial mutant (Jørgensen *et al.*, 1992; Dreiseitl, 2012; http://www.crpmb.org/mlo/). The *mlo-11* mutation results in aberrant transcription and thus a greatly reduced level of the MLO protein (Piffanelli *et al.*, 2004). *mlo*-mediated resistance to *B*. *graminis* f. sp. *hordei* is compromised by mutations to two genes designated *ROR1* or *ROR2* (*Required for mlo Resistance*; Freialdenhoven *et al.*, 1996).

There are agronomic costs associated with the use of *mlo* alleles in barley cultivars. Leaves of lines carrying *mlo* mutations typically suffer from apparently spontaneous necrotic spotting (Wolter *et al.*, 1993). This results in a yield penalty, the size of which is correlated with the amount of spotting, which implies that it should be possible to select *mlo* varieties in which the yield penalty is mitigated by reassortment of other, unidentified genes (Kjaer *et al.*, 1990). The second cost of using *mlo* mutations in spring barley is an enhanced susceptibility to several facultative pathogens, including *Magnaporthe oryzae* (blast; Jarosch *et al.*, 1999), *Cochliobolus sativus* (spot blotch; Kumar *et al.*, 2001), and *Fusarium graminearum* (head blight; Jansen *et al.*, 2005). Makepeace (2006) reported increased *Pyrenophora teres* (net blotch) in *mlo* plants although Jansen *et al.* (2007) reported no significant effect on this disease. By contrast, *mlo* may somewhat reduce susceptibility to *Rhynchosporium commune* (Makepeace, 2006; Makepeace *et al.*, 2007). Why mutations in *Mlo* increase susceptibility to most facultative pathogens tested is not yet understood.

Emerging pathogens are a constant threat to many crops and pose novel disease resistance problems to plant breeding programmes. Barley is the fourth most important cereal in terms of global production, across temperate regions in both the northern and southern hemispheres. Ramularia leaf spot (RLS), caused by the ascomycete fungus *Ramularia collo-cygni*, has been increasing in importance as a pathogen of this crop plant since the late 1990s (Walters *et al.*, 2008). *R*. *collo-cygni* is seed-borne and is also dispersed by spores (Schützendübel *et al.*, 2008; Havis *et al.*, 2011). Symptoms of RLS consist of characteristic reddish-brown rectangular lesions that are visible on both sides of the leaf and are often surrounded by a chlorotic halo. The disease is typically observed late in the growing season and can result in direct yield losses of up to 40% (Oxley and Havis, 2004) as well as greater losses in the market for malting because of higher screenings. There is a long latent period following *R*. *collo-cygni* infection before symptoms develop, implying that this fungus may be an endophyte that under certain conditions undergoes a transition to become a necrotrophic pathogen (Salamati and Reitan, 2006; Newton *et al.*, 2010; Havis *et al*., 2014). The reasons why RLS is increasing in importance are not known. Changes in agricultural practices and reduced fungicide sensitivity (Fountaine and Fraajie, 2009) may be involved in the rise of RLS in Europe and it has been suggested that changes in climate may have increased its prevalence (Roos *et al.*, 2011; West *et al.*, 2012).

The enhanced susceptibility to facultative fungal pathogens of plants containing mutations in *Mlo* (Jarosch *et al.*, 1999; Kumar *et al.*, 2001; Jansen *et al.*, 2005), the widespread use of spring barley varieties with *mlo* mildew resistance (http://www.crpmb.org/mlo/) and the emergence of RLS as an important new facultative disease of barley led to the hypothesis that *mlo* alleles may be implicated in the increased prevalence of RLS in Europe (Makepeace *et al.*, 2007). There have been contrasting reports about the effect of *mlo* mutations on the severity of RLS. In a series of field trials, adult plants of lines containing *mlo* mutations had lower RLS scores than near-isogenic lines (NILs) with the wild-type *Mlo* ^+^ allele. In experiments using artificial inoculation of seedlings in controlled environment conditions, however, plants with the *mlo-5* allele developed more RLS symptoms than *Mlo* ^+^ NILs, especially when the plants were exposed to high light intensity before inoculation (Brown and Makepeace, 2009). In inoculated field trials, commercial spring barley varieties with an *mlo* allele were on average more susceptible to RLS than *Mlo* ^+^ varieties, with all the most susceptible varieties having an *mlo* allele and all the least susceptible having *Mlo* ^+^ (Pinnschmidt and Sindberg, 2009); these results are consistent with the seedling experiments of Brown and Makepeace (2009). It is therefore possible that *mlo* mildew resistance may play an important role in the aetiology of RLS, a disease which has become a major threat to barley production in temperate regions. The evidence for such a relationship, however, is currently ambiguous.

This paper reports a genetic analysis of resistance to RLS, showing that *mlo* mutant alleles enhance susceptibility to RLS in spring barley in an interaction which appears to be environmentally dependent. Furthermore, experiments using barley lines with mutations in *ROR* genes indicate that the enhanced susceptibility to RLS is functionally linked to *mlo*-mediated mildew resistance. This implies that there is a trade off between resistance and susceptibility to these two diseases which depends on genetic background and environmental conditions. The data presented here emphasize the risk that susceptibility to previously unknown plant diseases can arise from plant breeding strategies that rely on the successful use of single major resistance genes to control one disease in elite crop varieties.

## Materials and methods

### Plant material

The genetics of resistance to RLS were investigated using two doubled-haploid (DH) populations developed from crosses between the spring barley cultivars Power×Braemar (POBR, 196 lines) and Decanter×Cocktail (DECO, 144 lines). In field trials, Power typically develops little RLS whilst Braemar is highly susceptible (Oxley *et al.*, 2008; Pinnschmidt and Sindberg, 2009; Oxley and Havis, 2010). Decanter and Cocktail have been described as resistant and susceptible, respectively, to RLS (Oxley and Havis, 2010). Decanter, however, is susceptible to RLS at the seedling stage (Makepeace *et al.*, 2008) and allows high *R*. *collo-cygni* DNA levels to build up despite limited symptom development in field conditions (Oxley and Havis, 2010). Both crosses allowed assessment of the effect of *mlo* on RLS development as Braemar and Decanter carry the mutant *mlo-11* allele while Power and Cocktail have the wild-type allele, *Mlo* ^+^.

In addition, *mlo* and *Mlo* ^+^ NILs were used to investigate the role of *mlo* alleles in susceptibility to *R*. *collo-cygni*. The backcross lines carrying *mlo-1*, *mlo-3*, *mlo-5*, and *mlo-9* mutations in cv. Ingrid, *mlo-1* in cv. Haisa, *mlo-3* in cv. Malteria Heda, and *mlo-5* in cv. Pallas (line P22) as well as *mlo-5*+*ror1-2* (line A89) and *mlo-5*+*ror2* (line A44) double mutants in the cv. Ingrid background have been described previously (Jørgensen, 1976; Kølster *et al.*, 1986; Freialdenhoven *et al.*, 1996).

### *Ramularia collo-cygni* isolate and culture medium

*R*. *collo-cygni* Rcc09B4 collected from Bush Estate, Midlothian, Scotland in 2009 by Dr Neil Havis (Scotland’s Rural College, Scotland) was used in all disease experiments. Fungal cultures were maintained as previously described (Makepeace *et al.*, 2008). Rcc09B4 liquid cultures were prepared as described by Peraldi *et al*. (2014).

### *Ramularia collo-cygni* inoculated seedling bioassays

Seeds were sown in Levington F2 compost media (Scotts Professional, Ipswich, UK) in a controlled environment room (Sanyo) with a 16/8 light dark cycle (220 μmol m^–2^ s^–1^ fluorescent lighting, 18/12 °C). In each of three replicate experiments, six seeds each from 196 lines of the POBR DH population plus the parents Power and Braemar were sown in rows in trays of 3×3×5cm (P60) pots. In separate experiments, 10 seeds of each of the barley near-isogenic *mlo* backcross lines and the Ingrid *mlo-5*+*ror* double-mutant lines were sown into individual 8×8×10cm pots. Rcc09B4 inoculum was prepared and plants inoculated with a slurry of hyphal fragments using an airbrush sprayer as described by Peraldi *et al*. (2014), developing the method of Makepeace *et al.* (2008). Plants were then placed under plastic covers to maintain high relative humidity (80–100%) in the dark for 48h, then under fluorescent lighting at 220 μmol m^–2^ s^–1^ for the duration of the experiment. Plants were watered as required to keep the potting medium moist but not saturated. To delay the onset of senescence of the prophyll, new leaf growth was cut from the plants every 2 or 3 days. Disease was scored as the percentage area of the prophyll covered with RLS lesions. Leaves were scored three to six times between 1 and 4 weeks after inoculation and the area under the disease progress curve (AUDPC; Shaner and Finney, 1977) was calculated. Pathology data for the *mlo* NILs and *ror* mutants was collected from separate experiments. Data on the *mlo* backcross lines was collected from three independent experiments and for the *ror* mutants from five experiments.

### Field trials of *Ramularia* leaf spot

Six field trials were conducted during 2012 at Irlbach, Mallersdorf and Moosburg an der Isar, Bavaria, Germany, and Bush (Midlothian), Glenrothes, and Perth, Scotland. Four replicates of each population were trailed in each country, with at least one complete replicate of each population at each site. Partial replication of lines within sites provided control of environmental variation, As well as the parents of each cross, 188 and 144 DH lines of the POBR and DECO populations, respectively, were tested. Field trial management, including cultivation, fertilizer, insecticide, and herbicide, was according to standard practice at each site. Where required, powdery mildew was controlled by spraying with Cyflamid (0.5 l ha^−1^), which does not affect RLS, and other diseases with a strobilurin (quinine-outside inhibitor) fungicide, to which *R. collo-cygni* is now resistant (Fountaine and Fraaije, 2009).

At each site, RLS was scored as percentage leaf area covered with disease symptoms on the highest leaf layer on which symptoms were fully developed but which had not yet senesced. At sites in Germany, this was the flag (topmost) leaf, but at Irlbach, scores were obtained for the flag leaf and the second leaf layer separately. RLS was scored on the second leaf layer at sites in Scotland. Plants in Germany were at growth stage (Tottman and Makepeace, 1979) 71–85 when assessed and those in Scotland at growth stage 67–72. RLS levels on the DECO population at Moosburg were very low and so this trial was not scored.

Several spotting syndromes affect barley (Oxley *et al.*, 2010). RLS symptoms were scored following Makepeace *et al.* (2008); see also Oxley *et al.* (2010, photographs on pp 9–16). Briefly, RLS was distinguished from other symptoms by ‘the four Rs’: small lesions which are rectangular, reddish-brown in colour, surrounded by a ring of chlorosis, and, as mature lesions, extend right through the leaf between the adaxial and abaxial surfaces. The spotting form of net blotch (*P. teres* var. *maculata*), with which RLS can be confused (Oxley *et al.*, 2010, p 50), was not observed in these field trials.

### Adult plant polytunnel trial

Adult plants of Ingrid, Haisa, Malteria Heda, and Pallas and *mlo* NILs of these cultivars were tested for susceptibility to RLS in a polytunnel trial. The experiment was planted in four blocks with three replicate plants of each line in each block in a randomized block design. Plants were inoculated with one of two doses of Rcc09B4 inoculum (2.5 or 10%, v/v, liquid culture in water supplemented with 0.01%, v/v, Tween 20), and at one of two times, growth stage 32 or growth stage 65 (i.e. before or after heading). Inoculum was applied using a CP3 knapsack sprayer (Cooper Pegler, Villefranche-sur-Saône, France). RLS severity was scored after flowering (growth stage 67–72) as described for the field trials.

### R. *collo-cygni* DNA quantification

*In planta R. collo-cygni* DNA was quantified by quantitative PCR (Taylor *et al.*, 2010) in DNA extracts from leaves of plants inoculated with Rcc09B4, sampled on the last scoring date in each experiment. Genomic DNA was extracted using a DNeasy Plant Mini Kit (Qiagen) following the manufacturer’s instructions and amplified in a CFX96 thermocycler (Bio-Rad) as previously described (Taylor *et al.*, 2010). DNA was quantified by comparison to a standard curve of known *R. collo-cygni* genomic DNA concentrations. Measurements were made from five independent samples of each line collected from three independent experiments.

### *B. graminis* f. sp. *hordei* inoculation experiments

Six seeds of each of the POBR DH lines plus their parents were sown in F2 compost in P60 trays and placed on outdoor benches at the John Innes Centre to allow natural infection by *B*. *graminis* f. sp. *hordei*. Plants were scored for mildew at growth stage 36 using the 0–4 infection-type scale (Moseman *et al.*, 1965). In practice, because only scores of 0 or 4 on the infection-type scale were recorded, they were converted to 0 and 1, respectively, to indicate absence or presence of mildew. Two independent experiments were done, which produced identical scores for all lines.

### Genotyping

The POBR and DECO DH populations, namely all 196 POBR lines and 144 DECO lines, were genotyped with the cultivar-optimized subset of 384 single-nucleotide polymorphism markers (Moragues *et al.*, 2010). Two additional PCR markers that distinguished between the wild-type *MLO* allele and the introgressed chromosome segment bearing *mlo-11* were added to confirm the *MLO* status of each line in the POBR population (Piffanelli *et al.*, 2004; Reinstadler *et al.*, 2010). Marker 11_20119 was used as a diagnostic marker for *mlo-11* in the DECO DH population. A genetic map of each cross was produced using the programme JoinMap version 3.0 (van Ooijen and Voorrips, 2001). Markers were formed into one or more groups within known chromosomal locations using a log-likelihood (LOD) threshold (details in Results). A map of the markers in each group was then estimated using the following parameters: minimum LOD to detect linkage 1.0, maximum recombination fraction 0.4, maximum LOD jump to retain marker in map 5.0. After the addition of each marker, the map was ‘rippled’ by optimizing the order of triplets of mapped markers. The Kosambi map function was used to transform recombination fractions to map distances. All chromosomes were mapped in the first round of the JoinMap method, indicating that there was little or no conflict in the map positions of linked markers.

### Statistical analysis

In all experiments, disease data were analysed as AUDPC as a percentage of the maximum possible AUDPC (maxAUDPC). Data were logit-transformed to normalize the variance of errors and make the errors independent of expected values. An empirical logit transformation (Collett, 2002), log_e_[(AUDPC+0.25)/(maxAUDPC−AUDPC+0.25)], was used to avoid an undefined transformed value when AUDPC=0 or 100, although the latter result did not occur in these experiments.

Seedling disease data were analysed by linear mixed modelling. To estimate the effect of *Mlo* alleles, the fixed effects were Experiment*Mlo genotype, the random effects were Line and Tray nested within (/) experiment, and the residual random term was Row within Tray. To estimate lines’ mean scores for quantitative trait locus (QTL) analysis, the fixed model was Experiment*Line and the random model Experiment/Tray/Row.

To analyse data from the *Mlo* NILs and the Ingrid *mlo-5*+*ror* lines, Experiment and Line were fixed effects and Pot the random effect. The significance of differences between lines or other factors, here and in other experiments, was tested by Fisher’s protected least significant difference. Data on amounts of log-transformed amounts of *R*. *collo-cygni* DNA in seedlings were analysed by general linear modelling. The model used was Line + Experiment.

Although all field trials were laid out as two incomplete blocks, the arrangement of blocks was not relevant to spatial variation at most trial sites. Predicted means for each line were estimated separately in each trial.

Post-hoc analysis of spatial layout was assessed in a model which fitted the two major spatial axes of the trial layout then the blocks. Blocks and directional axes were dropped successively from the model if they were not statistically significant (*P*>0.05) in a general linear model. The effect of Line at each site was estimated in a model with relevant spatial variables+Line as fixed effects and Plot as the random effect. The effect of *Mlo* allele was estimated using spatial variables + *Mlo* as fixed effects and Line + Plot as random effects.

In the inoculated polytunnel trial of the NILs as adult plants, treatment factors + Line were fixed effects and Replicate the random effect.

Where required, the significance of differences in unplanned comparisons of different factors was tested by Fisher’s protected least significant difference. All statistical analysis was conducted using Genstat version 15 (Payne *et al.*, 2009).

### QTL analysis

Predicted means of logit-transformed percentage RLS for each line were used to identify and map QTL for susceptibility to RLS, using the programme MapQTL 5 (van Ooijen, 2004). Data from each population (POBR or DECO) in each trial, including seedling experiments on POBR, were analysed separately. For each dataset, automatic cofactor selection with a significance test probability (*P*) of 0.02 was run, first for each linkage group separately then iteratively for the whole genome, as described in the MapQTL 5 manual. Once a final set of cofactors was selected, QTLs were located by multiple QTL mapping. The significance of mapped QTLs was determined by a permutation test (Churchill and Doerge, 1994). To test the prior hypothesis of an association between *Mlo* alleles and severity of RLS, the significance threshold for chromosome 4H was used. The significance of other QTLs was tested by the higher, genome-wide *P*-value.

## Results

### *Ramularia* leaf spot in field trials and seedling experiments

**Fig. 1.**
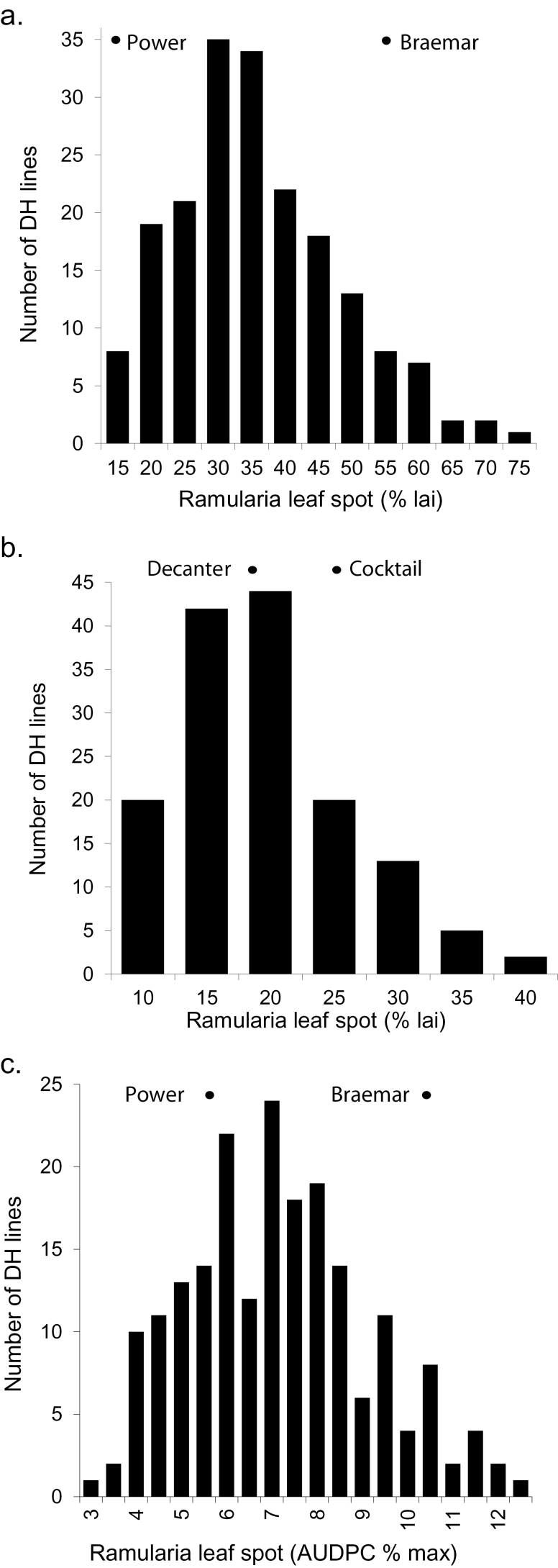
Histograms of the mean Ramularia leaf spot disease levels in field trials of the doubled-haploid (DH) populations: Power×Braemar (a), Decanter×Cocktail (b), and seedlings of Power×Braemar (c). Arrows indicate the means for the parents of each population. lai, leaf area infected.

**Fig. 2.**
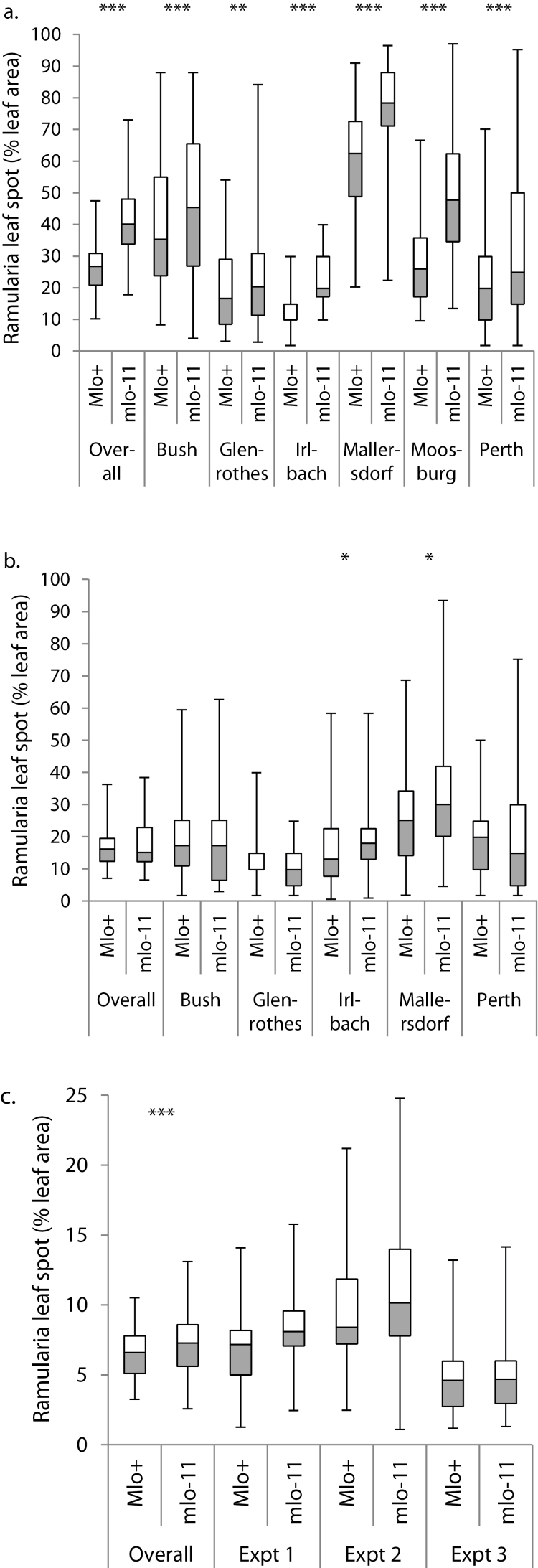
Boxplots showing the effects of *mlo* on Ramularia leaf spot development in field trials of the doubled-haploid (DH) populations: Power×Braemar (a), Decanter×Cocktail (b), and seedlings of Power×Braemar (c). The effects of *mlo* on RLS symptom severity in six individual field environments for both populations and RLS area under the disease progress curve (% max) in three independent inoculated seedling experiments of the Power×Braemar doubled-haploid population as well as the overall means are shown. The allelic state of *mlo* in the Power×Braemar population was determined by the *mlo11* marker MITE (Reinstadler *et al.*, 2010) and by marker 11_20119 in the Decanter×Cocktail population.

Mean RLS severity on each line of both DH populations had an approximately continuous distribution, both within trials and across the series of trials. The range of mean RLS severity was larger for the POBR population (Fig. 1a) than for DECO (Fig. 1b), reflecting a substantially greater difference between Power (12%) and Braemar (53%) than between Decanter (18%) and Cocktail (24%). The *Mlo* allele had a large, significant effect on RLS scores in the POBR population (*P*<0.001), with a mean score for the *Mlo* ^+^ lines of 22.6% (95% confidence interval, CI, 21.3–24.0%) and 35.3% (95% CI 33.5–37.3%) for the *mlo11* lines. There was a small but significant interaction between the *Mlo* allele and the field trial site (*P*<0.001); while there was significantly more RLS on *mlo-11* lines at all six sites, this effect was stronger in the trials in Germany than in Scotland (Fig. 2a). In the DECO population, the *Mlo* allele did not have a significant effect on RLS scores in the series of trials as a whole (*P*=0.6) but there was a significant interaction with trial site (*P*=0.002). At both sites scored in Germany, *mlo-11* lines had significantly more RLS than *Mlo* ^+^ lines, whereas at all three sites in Scotland, the *mlo-11* lines had less RLS than *Mlo* ^+^ but the differences were not significant (*P*>0.1 in each case, Fig. 2b). These results imply that both the environment and the genetic background influence the effect of *Mlo* alleles on RLS.

The POBR population was also tested for responses to *R*. *collo-cygni* at the seedling stage. As in the field trials, RLS severity in seedlings of the POBR lines had an approximately continuous distribution (Fig. 1c). Disease severity varied between experiments (*P*<0.001) but in all three, the more-resistant parent Power had less RLS than Braemar. The presence of the wild-type *Mlo*+ allele significantly reduced RLS levels in the seedling assays (*P*<0.001), a trend that was consistent across experiments (*P*=0.09, Fig. 2c). The mean of the AUDPC of RLS was 6.4% (95% CI 5.8−7.0%) for *Mlo* ^+^ and 7.4% (95% CI 6.8−8.1%) for *mlo-11*.

### QTL analysis of RLS severity

Of the 384 single-nucleotide polymorphism markers tested, 125 and 127 were polymorphic between the parents of the POBR and DECO DH populations, respectively, and 122 and 120 markers were mapped in these populations. With one exception, linkage groups were formed at a threshold of LOD≥4. In the POBR population (Supplementary Fig. S1, available at *JXB* online), there was a spurious linkage between two markers, one previously assigned to chromosome 2H and the other to 6H, so those groups were separated at LOD≥6. Chromosomes 1H and 2H were mapped in two linkage groups each. One marker, previously assigned to chromosome 4H, had strongly distorted segregation and was not included in the map while two other markers, 11_21353 and 11_21504, were omitted because, while they had previously been mapped to the middle of chromosome 4, in POBR they were closely linked to each other but not to other markers on chromosome 4. As expected, the two *Mlo*-specific markers cosegregated and mapped to the distal end of the long arm of chromosome 4H. In DECO (Supplementary Fig. S2), which was a smaller population, the only chromosome with a single linkage group was 4H. Seven markers, dispersed in the genome, were excluded because they had distorted segregation.

A single QTL for RLS severity in field trials was closely linked to the *Mlo* locus in the POBR population, with increased resistance contributed by the allele from Power, which has the *Mlo* ^+^ allele for mildew susceptibility. This QTL was significant (*P*<0.05) at five of the six trial sites with narrow-sense heritability (equivalent to the percentage genetic variation in disease severity explained by the QTL) between 4 and 37% (Table 1). The *Mlo* locus had a greater effect on RLS at the sites in Germany than in Scotland (Table 1 and Fig. 2a). No QTL for RLS severity associated with the BOPA1 marker 11_20119, closely linked to *Mlo*, was identified in the DECO population at any field trial site, which is consistent with the smaller and less consistent effect of *Mlo* alleles on RLS in this population (Fig. 2b). Analysis of the seedling data also identified a QTL for RLS severity at the *Mlo* locus. Although *mlo-11* lines had a greater mean RLS score in all three experiments, the QTL was only significant (*P*<0.05) in one experiment, in which the narrow-sense heritability associated with *mlo-11* was 6% (Table 1).

**Table 1.** QTL analysis of *Ramularia* leaf spot scores in a population of F1 doubled-haploid progeny of the spring barley cultivars Power×BraemarLog-likelihood (LOD) scores were calculated by multiple QTL mapping (details in text) of logit-transformed percentage data on leaf area affected by *Ramularia* leaf spot (RLS). LOD are shown for four markers at the distal end of chromosome arm 4HL; no QTL were detected in other regions of the genome. RLS: seedling, maximum area under the disease progress curve; field trials, leaf covered with RLS. Additive effect calculated on logit scale; *h* ^2^, narrow-sense heritability.

Site	LOD (position on 4H, cM)				Critical LOD^*a*^	RLS for allelic class (%)		Additive effect	*h* ^2^ (%)
	11_10751 (100.5)	11_20732 (102.1)	11_20119 (113.1)	MLO (115.7)		Power	Braemar		
Seedling 1	0.3	1.1	2.5	2.4	1.6	6.4	7.7	0.095	5.6
Seedling 2	0.3	0.1	0.6	1.0	1.5	8.8	10.2	0.075	2.3
Seedling 3	0.4	0.0	0.3	0.1	1.6	4.2	4.5	0.040	0.6
All field trials	0.1	0.2	15.3	15.9	1.8	26.2	40.1	0.315	31.1
Germany	0.1	0.4	17.1	18.0	1.7	29.6	49.1	0.413	34.5
Irlbach	0.0	0.1	17.9	19.4	1.6	11.0	21.1	0.380	36.6
Mallersdorf	0.1	5.1	6.6	6.3	1.5	63.0	78.6	0.381	14.8
Moosburg an der Isar	0.0	0.1	11.5	12.7	1.5	27.0	50.4	0.502	25.8
Scotland	0.0	2.2	3.7	3.6	1.6	23.0	31.5	0.214	8.2
Bush	0.0	0.0	1.3	1.8	1.7	37.7	47.7	0.202	4.2
Glenrothes	0.1	0.9	1.0	0.6	1.6	16.3	20.6	0.144	2.4
Perth	0.0	0.1	2.9	3.0	1.6	16.2	29.3	0.380	7.6

^*a*^ *P*≤0.05 from a permutation test (van Ooijen, 2004).

No other QTL were detected above the critical LOD threshold in either the field trials or seedling experiments when RLS data for all the progeny of the POBR population were analysed. Data for *Mlo* ^+^ and *mlo-11* lines were analysed separately in POBR, in case the effect of *Mlo* was epistatic to that of other genes. Although some QTL effects were significant at individual sites, none were replicated at more than one site. It is quite likely that their identification was a type I statistical error (false positive). The absence of consistent QTLs in the subdivided data implies that no epistatic effect on RLS of other genes interacting with *Mlo* alleles was identified. In the DECO population, a minor QTL (LOD=3.1) was identified at Perth near 11_20713 on chromosome 5H; mean scores were 10 and 18% for lines with the Decanter and Cocktail alleles, respectively

To test the function of *mlo-11* on conferring almost-complete immunity to powdery mildew, the POBR DH population was screened for resistance to natural infection by *B*. *graminis* f. sp. *hordei*. Two QTL for mildew resistance were identified. One, on chromosome 4H, coincided with the *mlo-11* allele in Braemar and accounted for 42% of genetic variation in mildew (Table 2). The other, controlling 13% of variation, was on chromosome 1H between markers 11_10332 and 11_10775, which flank the multiallelic *Mla* gene controlling gene-for-gene resistance to mildew. As *mlo-11* confers strong resistance to mildew and is thus epistatic to other mildew-resistance genes, the QTL analysis was repeated for the *Mlo* ^+^ lines only; the QTL on chromosome 1 was now the only one identified and controlled 39% of genetic variation (Table 2). This implies that Braemar has an unidentified allele of *Mla* effective against the local population of *B. graminis* f. sp. *hordei*.

**Table 2.** QTL analysis of powdery mildew scores in a population of F1 doubled-haploid progeny of the spring barley cultivars Power×BraemarLog-likelihood (LOD) scores were calculated by multiple QTL mapping of data on the presence or absence of mildew on barley seedlings exposed to natural inoculum. LOD are shown for relevant markers around the two QTL detected on chromosomes 1H and 4H. *h* ^2^: narrow-sense heritability.

Chromosome	Marker	Position (cM)	LOD^a^	Mean score for allelic class		*h* ^2^ (%)
				Power	Braemar	
All lines						
1H	11_21226	6.2	0.0			
	11_10332	10.5	8.9			
	11_10775	14.1	10.2	0.51	0.17	13.0
	11_10030	16.2	0.2			
4H	11_10262	62.5	0.3			
	11_10751	102.0	2.2			
	11_20732	103.5	1.1			
	11_20119	114.8	22.8			
	*Mlo*	117.3	26.4	0.65	0.03	41.6
*Mlo* ^*+*^ lines						
1H	11_21226	6.2	0.2			
	11_10332	10.5	9.2			
	11_10775	14.1	10.9	0.96	0.36	39.1
	11_10030	16.2	0.4			

^*a*^Genome-wide critical value of LOD for unplanned tests = 2.7 (*P* ≤ 0.05, permutation test: van Ooijen, 2004).

### *Ramularia* leaf spot in *Mlo* near-isogenic lines

**Fig. 3.**
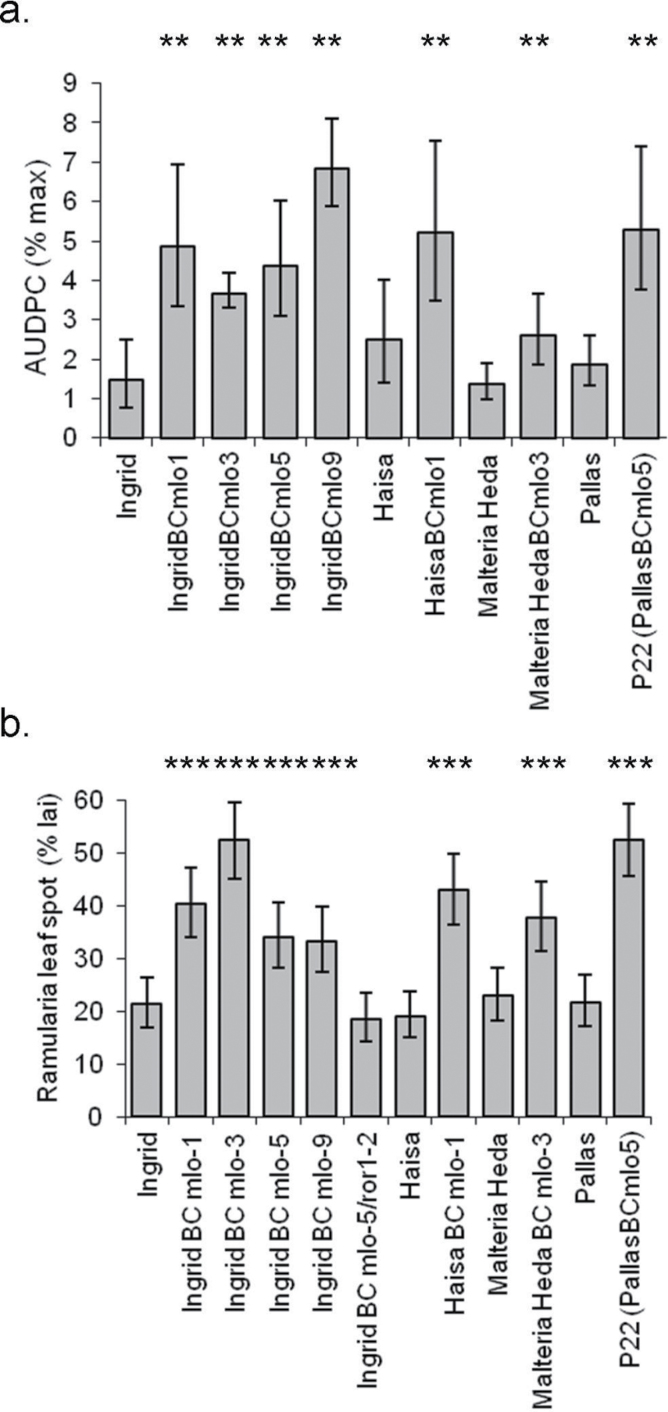
Ramularia leaf spot development on near-isogenic *mlo* mutant lines in controlled environment room seedling assays (a) and in inoculated adult plants (b). The effects of independent *mlo* mutations in different barley genetic backgrounds on *Ramularia* leaf spot area under the disease progress curve (AUDPC) from three independent inoculated seedling experiments and on *Ramularia* leaf spot symptom severity from a single inoculated adult plant test are shown.

In seedlings of NILs inoculated with *R*. *collo-cygni* isolate Rcc09B4, the presence of mutant *mlo* alleles was associated with significantly greater RLS in all the genetic backgrounds tested compared to mother lines with the wild-type *Mlo* ^+^ allele (*P*<0.01; Fig. 3a). The presence of the *mlo* mutant alleles also significantly increased RLS symptoms on inoculated adult plants compared to their respective wild-type lines (*P*<0.001; Fig 3b). There was also an effect of the timing and dose of inoculation on development of RLS in the adult-plant experiment, with greater disease on plants inoculated with the higher dose after heading than in the other treatments (*P*<0.01). There were significant differences in RLS levels between seedlings (*P*<0.01) and adult plants (*P*<0.001) of the Ingrid NILs with different *mlo* mutant alleles but these differences were not consistent between the two plant growth stages (Fig. 3).

### Effect of Ror alleles on *Ramularia* leaf spot and fungal DNA in *mlo-5* plants

**Fig. 4.**
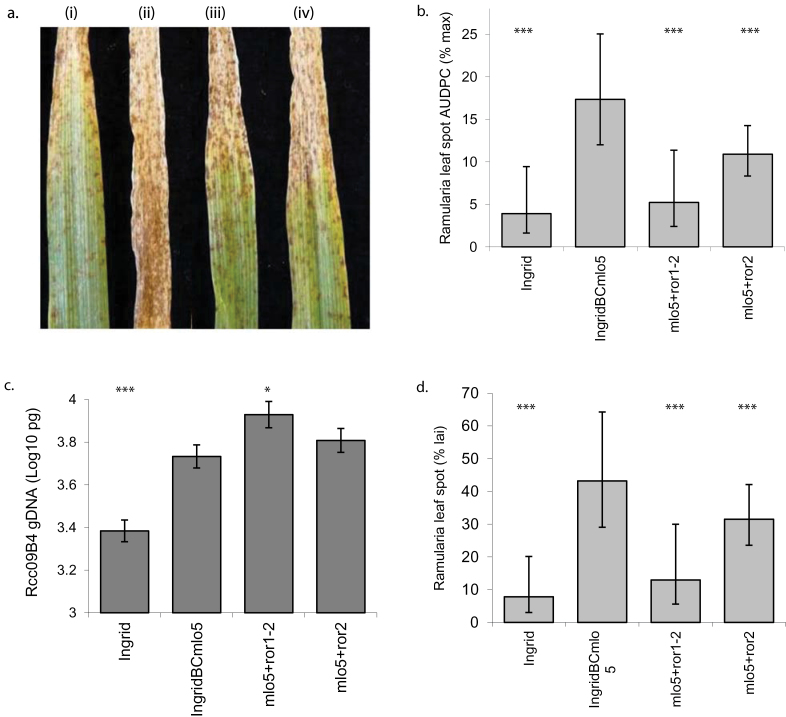
Mutations at the *ROR1* and *ROR2* loci reduce *mlo-5*-mediated enhanced Ramularia leaf spot susceptibility but do not reduce associated *Ramularia collo-cygni in planta* DNA levels. (a) Ramularia leaf spot disease symptoms on Ingrid (i), IngridBC*mlo-5* (ii), *mlo-5*+*ror1-2* (iii), *mlo-5*+*ror2* (iv). (b) Ramularia leaf spot area under the disease progress curve (AUDPC) development in inoculated seedling trails. (c) Quantitative PCR analysis of *R*. *collo-cygni* DNA levels in inoculated leaves sampled at the last score date of each experiment. (d) Final Ramularia leaf spot disease levels at last score date. Data are from three independent experiments.

Susceptibility to RLS was tested in NILs of cv. Ingrid with the *mlo*-5 allele in which susceptibility to mildew has been partially restored by mutations at the *ROR1* or *ROR2* loci (Freialdenhoven *et al.*, 1996). Seedlings with the *mlo-5+ror1-2* or *mlo-5*+*ror2* genotypes had significantly less RLS symptoms than near-isogenic IngridBC*mlo-5* plants with wild-type *ROR1* and *ROR2* (*P*<0.001, Fig. 4a and b). The *mlo-5*+*ror1-2* genotype had less disease than the *mlo-5*+*ror2* genotype (Fig. 4b). In inoculated adult plants, RLS symptoms on *mlo-5*+*ror1-2* plants were reduced approximately to the level on cv. Ingrid (Fig. 3b).

*R*. *collo-cygni* DNA levels were measured by quantitative PCR in leaves of Ingrid, IngridBC*mlo-5*, and the two *mlo-5*+*ror* genotypes at the final scoring date of each experiment to test if RLS symptoms were correlated with fungal biomass. Fungal DNA levels were higher in IngridBC*mlo-5* than in Ingrid (*P*<0.001, Fig. 4c), consistent with RLS symptoms in these two genotypes (Fig. 4b). *R*. *collo-cygni* DNA levels in *mlo-5*+*ror1-2* and *mlo-5*+*ror2* plants were not reduced compared to IngridBC*mlo-5* (Fig. 4c) despite the reduced disease development and lower final RLS levels in these mutants (Fig. 4b and d). *R*. *collo-cygni* DNA levels were similar between IngridBC*mlo-5* and *mlo-5*+*ror2* but were significantly higher in *mlo-5*+*ror1-2* (*P*<0.05, Fig. 4c).

## Discussion

These results show that recessive loss-of-function alleles of *Mlo*, which confer resistance to powdery mildew (Büschges *et al.*, 1997), greatly increase susceptibility of barley to Ramularia leaf spot caused by *R. collo-cygni*. This effect is apparent in controlled, inoculated experiments on near-isogenic lines in which different *mlo* alleles are present in various genetic backgrounds (Fig. 3). It is also associated with the introgression of *mlo-11* in barley populations in naturally infected field trials (Fig. 2a and Table 1) but the effect of *mlo-11* on increasing susceptibility to RLS depends on the genetic background (Fig. 2a and b) and the environment (compare sites within Fig. 2a and b).

The effect of *mlo-11* on increasing RLS in the POBR population was strong with a QTL for RLS mapping to the *Mlo* locus (Table 1). Although the single-nucleotide polymorphism markers 11_20119, which is closely linked to *mlo-11* (Table 1) was associated with higher RLS in the DECO population at the sites in Germany (but not Scotland), the effect was smaller than for POBR and there was no significant QTL effect at that locus (note that the significance test in QTL mapping is more conservative than the *F*-test in linear mixed models).

The conclusion that the effect of *mlo-11* on RLS depends on environmental conditions is consistent with previous results. Abiotic stress such as exposure to high light intensity increases RLS symptoms (Makepeace *et al.*, 2008; Peraldi *et al*., 2014) and this response is enhanced by the presence of *mlo* alleles (Brown and Makepeace, 2009). The result that, in inoculated adult plants, all *mlo* alleles tested in diverse genetic backgrounds were consistently associated with increased RLS (Fig. 3) is in striking contrast to Makepeace *et al.* (2007), who found that, in the same lines, *mlo* alleles were associated with somewhat lower RLS symptoms in trials in Scotland and Ireland. Two major differences between the present results and those of Makepeace *et al.* (2007) are that the polytunnel inevitably provides environmental conditions which are unnatural and may be stressful and that levels of RLS were considerably greater in the polytunnel. Either or both factors may have led to the contrasting results.

Developing elite crop varieties that maintain high yields across different environments is an important goal for plant breeding. Durable disease resistance helps to increase the stability and predictability of yield in the face of diverse pathogens. While many durable resistances are oligogenic or polygenic (St Clair, 2010), those controlled by single genes with large effects are particularly easy to select and are therefore attractive for breeding. *mlo* resistance to powdery mildew is an outstanding example of such a gene for durable resistance as it has been effective since its introduction into spring barley cultivars in the 1970s (Jørgensen *et al.*, 1992), especially since the early 1990s (Thomas *et al.*, 1998; Dreiseitl, 2012). Biotrophic parasites such as mildew fungi are closely adapted to their hosts’ physiology so a mutation which involves almost complete loss of susceptibility to such a pathogen might be expected to have substantial pleiotropic effects on the plant. *mlo* mutations incur agronomic costs caused by spontaneous leaf spotting reflected in a yield penalty (Kjaer *et al.*, 1990; Thomas *et al.*, 1998) but even so, more than half of all northern European spring cultivars now have an *mlo* mildew resistance allele (Dreiseitl, 2012; http://www.crpmb.org/mlo/). *mlo* alleles also elevate susceptibility to several hemibiotrophic fungi in laboratory experiments (Jarosch *et al.*, 1999; Kumar *et al.*, 2001; Jansen *et al.*, 2005), although those studies did not investigate the implications of increased susceptibility for crop management.

This paper demonstrates that loss-of-function and reduced-function mutants of *Mlo* are associated with increased susceptibility to a facultative disease in field conditions. Spot blotch (*C. sativus*) and blast (*M. oryzae*) are significant pathogens in the warm tropics, where powdery mildew is rare and *mlo* mutations are not used in breeding. RLS, by contrast, has become an important challenge to economic production of barley in many regions in temperate latitudes over the last 15 years, including Europe, New Zealand, and the Southern Cone of South America (Walters *et al.*, 2008), all places where *mlo* alleles, particularly *mlo-11*, have been important in breeding for durable resistance to mildew in spring barley. The implication of the results presented here is that, along with climatic and agronomic factors (Oxley and Havis, 2010; Roos *et al.*, 2011; West *et al.*, 2012), widespread use of *mlo* alleles in spring barley cultivars may have contributed to the epidemic of RLS. Indeed, given that the effect of *mlo* on RLS depends on environmental conditions, it is conceivable that the widespread use of *mlo-11* may have interacted with changes in agronomy and the climate to stimulate the RLS epidemic.

The effect of *mlo* alleles on increasing RLS may be mediated by perturbation of reactive oxygen species (ROS). RLS is a late season disease with symptoms typically occurring after ear emergence (Oxley and Havis, 2004), implying that the fungus maintains a relatively benign association with its host until one or more factors trigger a transition from endophyte to necrotrophic pathogen. Some endophytes become pathogenic in response to specific environmental and host physiological stimuli (Schulz and Boyle, 2005; Kogel *et al.*, 2006). In the field, the onset of RLS symptoms is preceded by a general decline in the host antioxidant system, while leaf senescence may promote the development of disease (Schützendübel *et al.*, 2008). As the host antioxidant system declines, the plant undergoes oxidative stress as levels of ROS increase. In experimental conditions, RLS symptom development is promoted by environmental stresses such as high light (Makepeace *et al.*, 2007; Brown and Makepeace, 2009) which increase ROS levels, promoting oxidative stress and leaf senescence (Biswal and Biswal, 1984; Mullineaux *et al.*, 2006).

The effect of *mlo* alleles on RLS may relate to leaf senescence, ROS levels, or both, consistent with the results of Schützendübel *et al.* (2008). *mlo* mutations do not affect the onset of leaf senescence but they accelerate the rate at which senescence progresses once it has been initiated (Piffanelli *et al.*, 2002). ROS production and the onset of host cell death are also altered in *mlo* plants (Peterhänsal *et al.*, 1997; Piffanelli *et al.*, 2002) and *mlo* resistance to *B*. *graminis* f. sp. *hordei* is associated with an H_2_O_2_ burst beneath the site of attempted penetration. Accelerated leaf senescence in *mlo* plants may trigger more rapid transition of *R*. *collo-cygni* from endophyte to necrotroph, which may be further enhanced by environmental conditions that induce oxidative stress in the plant. Enhanced mesophyll cell death has been suggested as the reason why *mlo* mutants are more susceptible to the hemibiotrophic pathogens *M*. *oryzae* and *Bipolaris sorokiniana* (Jarosch *et al.*, 1999; Kumar *et al.*, 2001) although restricted cell death in a barley gain-of-function DELLA mutant increased RLS symptom development whereas the loss-of-function mutant with enhanced cell death was more resistant to this disease (Saville *et al.*, 2012). These results may not be inconsistent with each other because restriction of host cell death during endophytic growth of *R*. *collo-cygni* may promote leaf colonization whereas enhanced mesophyll cell death once the fungus is established may increase RLS symptom development. The latter effect may be responsible for increased RLS in *mlo* plants.

*mlo*-mediated resistance to *B. graminis* f. sp. *hordei* is a prepenetration response preventing fungal entry into the attacked epidermal cell (Piffanelli *et al.*, 2002), which requires the genes *ROR1* and *ROR2* to be functional. Whereas the susceptibility of *mlo-5*+*ror1-2* and *mlo-5*+*ror2* lines to mildew is partially restored compared to *mlo-5 ROR1 ROR2* lines (Freialdenhoven *et al.*, 1996), the opposite effect on RLS was detected (Fig. 4), implying that *mlo*-dependent enhanced susceptibility to the necrotroph *R. collo-cygni* may operate by the same pathway as *mlo*-mediated resistance to the biotrophic *B. graminis* f. sp. *hordei*. While the *ror* mutants reduced the development of RLS symptoms, they did not reduce the amount of the *R. collo-cygni* fungus in barley leaves (Fig. 4c), implying that the *ROR* genes affect transition from the endophytic phase of the fungal life cycle to necrotrophy but not the growth of the fungus itself. The peroxide burst in response to *B. graminis* f. sp. *hordei* is enhanced in *mlo* mutants and extends into the mesophyll resulting in cell death (Peterhänsal *et al.*, 1997; Piffanelli *et al.*, 2002). Mutation of either *ROR1* or *ROR2* diminishes this peroxide burst but both *mlo-5 ror* mutant genotypes accumulate more H_2_O_2_ in the mesophyll and experience more mesophyll cell death than in *mlo-5 ROR1 ROR2* (Huckelhoven *et al.*, 2000; Piffanelli *et al.*, 2002). H_2_O_2_ accumulation may also be involved in the pathogenesis of *Bipolaris sorokiniana* on barley (Kumar *et al.*, 2001). A hypothesis arising from the work reported here, therefore, is that ROS, especially peroxide ions, stimulate the endophyte–necrotroph transition in *R. collo-cygni* but do not affect growth of the fungus itself *in planta*. The effect of *mlo* on RLS may be mediated directly by ROS as signalling molecules or indirectly via accelerated leaf senescence.

*mlo* mutations may promote susceptibility to different hemibiotrophic and necrotrophic pathogens by at least partly different mechanisms. As in RLS (Fig. 4c), enhanced development of blast symptoms in *mlo* plants is associated with enhanced growth of *M*. *oryzae* (Jarosch *et al.*, 1999) but *mlo*-promoted development of *M*. *oryzae* was not altered in the *mlo-5*+*ror1-2* mutant, unlike RLS (Fig. 4c) and *B*. *sorokiana* culture filtrate (Jarosch *et al.*, 1999; Kumar *et al.*, 2001).

The results here indicate that plant breeders should be able to combine *mlo-11* mildew resistance and polygenic partial resistance to RLS in spring barley cultivars. There was substantial genetic variation in susceptibility to RLS in both *Mlo* ^+^ and *mlo-11* lines in both crosses (Figs. 1 and 2) although no other QTL controlling significant proportions of resistance to RLS was identified (Table 1). Given a method of selecting reliably for reduced susceptibility, breeders should be able to accumulate polygenes to control RLS in barley cultivars carrying *mlo-11* and there is considerable variation in RLS susceptibility amongst the highly mildew-resistant (mainly *mlo-11*) varieties on the UK Spring Barley Recommended List (http://www.hgca.com/content.template/23/0/Varieties/Varieties/Varieties%20Home%20Page.mspx). This could be investigated genetically in crosses between *mlo-11* cultivars such as Braemar and Decanter, which differ greatly in susceptibility to RLS. Genome-wide association studies could assist the search for genes which control RLS in *mlo* cultivars. Care would need to be taken to trial these populations in diverse environments to account for the environmental sensitivity of the *mlo* effect on RLS.

The emergence of *Ramularia* leaf spot as a major new disease of barley in temperate regions, including much of Europe since the mid-1990s (Walters *et al.*, 2008) may have been stimulated by the *mlo* mildew resistance in spring barley in combination with agricultural and meteorological factors. In laboratory experiments, *mlo* alleles have also been associated with susceptibility to two other economically significant diseases, *Fusarium* head blight (Jansen *et al.*, 2005) and net blotch (*P. teres*; Makepeace, 2006). The availability of *mlo* mutations to control mildew has benefitted barley production but, as there are effective alternative methods of controlling mildew, including several systemic fungicides (Walters *et al.*, 2012) and breeding for durable, partial resistance (St Clair, 2010), it is possible that the costs of using *mlo* alleles have outweighed their benefits. It may be possible, however, to restore the value of *mlo* in breeding by combining it with partial resistance to RLS and, if necessary, net blotch and *Fusarium* head blight. The existence of substantial genetic variation in RLS severity, over and above the susceptibility conferred by *mlo-11* (Fig. 2), implies that there is scope for selection of cultivars which combine strong *mlo* resistance to mildew with adequate resistance to RLS.

## Supplementary material

Supplementary data are available at *JXB* online.

Supplementary Fig. S1. Genetic map of the Power×Braemar doubled-haploid population

Supplementary Fig. S2. Genetic map of the Decanter×Cock tail doubled-haploid population

Supplementary Data

## References

[CIT0001] Biswal UC Biswal B 1984 Photocontrol of leaf senescence. Photochemistry and Photobiology 39, 875–879

[CIT0002] Brown JKM. Makepeace JC 2009 The effect of genetic variation in barley on responses to *Ramularia collo-cygni* . Aspects of Applied Biology 92, 43–47

[CIT0003] Brown JKM Rant JC 2013 Fitness costs and trade-offs of disease resistance and their consequences for breeding arable crops. Plant Pathology in press (10.1111/ppa.12163).

[CIT0004] Büschges R Hollricher K Panstruga R 1997 The barley *mlo* gene: a novel control element of plant pathogen resistance. Cell 88, 695–705 9054509 10.1016/s0092-8674(00)81912-1

[CIT0005] Churchill GA Doerge RW 1994 Empirical threshold values for quantitative trait mapping. Genetics 138, 963–971 7851788 10.1093/genetics/138.3.963 PMC1206241

[CIT0006] Collett D 2002 Modelling binary data , 2nd ed. Boca Raton, USA: Chapman and Hall/CRC

[CIT0007] Consonni C Humphry ME Hartmann HA 2006 Conserved requirement for a plant host cell protein in powdery mildew pathogenesis. Nature Genetics 38, 716–720 16732289 10.1038/ng1806

[CIT0008] Dreiseitl A 2012 Frequency of powdery mildew resistances in spring barley cultivars in Czech variety trials. Plant Protection Science 48, 17–20

[CIT0009] Fountaine JM Fraaije BA 2009 Development of QoI resistant alleles in populations of *Ramularia collo-cygni* . Aspects of Applied Biology 92, 123–126

[CIT0010] Freialdenhoven A Peterhänsal C Kurth J Kreuzaler F Schulze-Lefert P 1996 Identification of genes required for the function of non-race-specific *mlo* resistance to powdery mildew in barley. The Plant Cell 8, 5–14 12239354 10.1105/tpc.8.1.5 PMC161077

[CIT0011] Havis ND Maguire K Knight SM Oxley SJP 2011 *Ramularia collo-cygni—*an increasing problem for barley growers in Soutehrn Britain. Aspects of Applied Biology 106, 131–136

[CIT0012] Havis ND Nyman M Oxley SJP 2014 Evidence for seed transmission and asymptomatic growth of *Ramularia collo-cygni* in barley (*Hordeum vulgare*). Plant Pathology in press ( 10.1111/ppa.12162).

[CIT0013] Huckelhoven R Trujillo M Kogel KH 2000 Mutations in *Ror1* and *Ror2* genes cause modification of hydrogen peroxide accumulation in *mlo*-barley under attack from the powdery mildew fungus. Molecular Plant Pathology 1, 287–292 20572975 10.1046/j.1364-3703.2000.00032.x

[CIT0014] Jansen C von Wettstein D Schafer W Kogel KH Felk A Maier FJ 2005 Infection patterns in barley and wheat spikes inoculated with wild-type and trichodiene synthase gene disrupted *Fusarium graminearum* . Proceedings of the National Academy of Sciences, USA 102, 16892–16897 10.1073/pnas.0508467102 PMC1283850 16263921

[CIT0015] Jansen M Jarosch B Schaffrath U 2007 The barley mutant *emr1* exhibits restored resistance against *Magnaporthe oryzae* in the hypersusceptible *mlo*-genetic background. Planta 225, 1381–1391 17143617 10.1007/s00425-006-0447-1

[CIT0016] Jarosch B Kogel KH Schaffrath U 1999 The ambivalence of the barley *Mlo* locus: mutations conferring resistance against powdery mildew (*Blumeria graminis* f. sp, *hordei*) enhance susceptibility to the rice blast fungus *Magnaporthe grisea* . Molecular Plant–Microbe Interactions 12, 508–514

[CIT0017] Jørgensen JH 1976 Identification of powdery mildew resistant barley mutants and their allelic relationship. In: Gaul H, editor, Barley genetics III. München: Karl Thiemig pp 446–455

[CIT0018] Jørgensen JH 1992 Discovery, characterization and exploitation of *Mlo* powdery mildew resistance in barley. Euphytica 63, 141–152

[CIT0019] Kim MC Panstruga R Elliott C Muller J Devoto A Yoon HW Park HC Cho MJ Schulze-Lefert P 2002 Calmodulin interacts with MLO protein to regulate defence against mildew in barley. Nature 416, 447–450 11919636 10.1038/416447a

[CIT0020] Kjaer B Jensen HP Jensen J Jorgensen JH 1990 Associations between 3 *ml-o* powdery mildew resistance genes and agronomic traits in barley. Euphytica 46, 185–193

[CIT0021] Kogel KH Franken P Huckelhoven R 2006 Endophyte or parasite—what decides? Current Opinion in Plant Biology 9, 358–363 16713330 10.1016/j.pbi.2006.05.001

[CIT0022] Kølster P Munk L Soglen O Løhde J 1986 Near-isogenic barley lines with genes for resistance to powdery mildew. Crop Science 25, 903–907

[CIT0023] Kumar J Huckelhoven R Beckhove U Nagarajan S Kogel KH 2001 A compromised *Mlo* pathway affects the response of barley to the necrotrophic fungus *Bipolaris sorokiniana* (teleomorph: *Cochliobolus sativus*) and its toxins. Phytopathology 91, 127–133 18944385 10.1094/PHYTO.2001.91.2.127

[CIT0024] Makepeace JC 2006 *The effect of the* mlo *mildew resistance gene on spotting diseases of barley* PhD thesis, Norwich, UK: Univeristy of East Anglia.

[CIT0025] Makepeace JC Havis ND Burke JI Oxley SJP Brown JKM 2008 A method of inoculating barley seedlings with *Ramularia collo-cygni* . Plant Pathology 57, 991–999

[CIT0026] Makepeace JC Oxley SJP Havis ND Hackett R Burke JI Brown JKM 2007 Associations between fungal and abiotic leaf spotting and the presence of *mlo* alleles in barley. Plant Pathology 56, 934–942

[CIT0027] Moragues M Comadran J Waugh R Milne I Flavell AJ Russell JR 2010 Effects of ascertainment bias and marker number on estimations of barley diversity from high-throughput SNP genotype data. Theoretical and Applied Genetics 120, 1525–1534 20157694 10.1007/s00122-010-1273-1

[CIT0028] Moseman JG Macer RCF Greeley LW 1965 Genetic studies with cultures of *Erysiphe graminis* f. sp. *hordei* virulent on *Hordeum spontaneum* . British Mycological Society Transactions 48, 479–489

[CIT0029] Mullineaux PM Karpinski S Baker NR 2006 Spatial dependence for hydrogen peroxide-directed signaling in light-stressed plants. Plant Physiology 141, 346–350 16760486 10.1104/pp.106.078162 PMC1475435

[CIT0030] Newton AC Fitt BDL Atkins SD Walters DR Daniell TJ 2010 Pathogenesis, parasitism and mutualism in the trophic space of microbe-plant interactions. Trends in Microbiology 18, 365–373 20598545 10.1016/j.tim.2010.06.002

[CIT0031] Oxley SJP Havis ND 2004 Development of *Ramularia collo-cygni* on spring barley and its impact on yield. In: Proceedings of the Dundee conference: crop protection in Northern Britain 2004. Dundee, UK: Association for Crop Protection in Northern Britain pp 147–152

[CIT0032] Oxley SJP Havis ND 2010 Managing Ramularia collo-cygni through varietal resistance, seed health and forecasting , HGCA project report. Kenilworth, UK: AHDB-HGCA

[CIT0033] Oxley SJP Havis ND Brown JKM Makepeace JC Fountaine J 2008 Impact and interactions of Ramularia collo-cygni and oxidative stress in barley , HGCA project report. Kenilworth, UK: AHDB-HGCA

[CIT0034] Oxley S Havis N Evans A Waterhouse S Tonguç L 2010 A guide to the recognition and understanding of Ramularia and other leaf spots of barley. Edinburgh, UK: SAC; Cheadle Hulme, UK: BASF

[CIT0035] Payne RW Murray DA Harding SA Baird DB Soutar DM 2009 GenStat for Windows , 12th ed. Hemel Hempstead, UK: VSN International

[CIT0036] Peraldi A Griffe LL Burt C McGrann GRD Nicholson P 2014 *Brachypodium distachyon* exhibits compatible interactions with *Oculimacula* spp. and *Ramularia collo-cygni*, providing the first pathosystem model to study eyespot and *Ramularia* leaf spot diseases. Plant Pathology in press (10.1111/ppa.12114). 10.1111/ppa.12114 PMC4480328 26146412

[CIT0037] Peterhänsal C Freialdenhoven A Kurth J Kolsch R Schulze-Lefert P 1997 Interaction analyses of genes required for resistance responses to powdery mildew in barley reveal distinct pathways leading to leaf cell death. The Plant Cell 9, 1397–1409 12237388 10.1105/tpc.9.8.1397 PMC157006

[CIT0038] Piffanelli P Ramsay L Waugh R Benabdelmouna A D’Hont A Hollricher K Jorgensen JH Schulze-Lefert P Panstruga R 2004 A barley cultivation-associated polymorphism conveys resistance to powdery mildew. Nature 430, 887–891 15318221 10.1038/nature02781

[CIT0039] Piffanelli P Zhou FS Casais C Orme J Jarosch B Schaffrath U Collins NC Panstruga R Schulze-Lefert P 2002 The barley MLO modulator of defense and cell death is responsive to biotic and abiotic stress stimuli. Plant Physiology 129, 1076–1085 12114562 10.1104/pp.010954 PMC166502

[CIT0040] Pinnschmidt HO Sindberg SA 2009 Assessing *Ramularia* leaf spot resistance of spring barley cultivars in the presence of other diseases. Aspects of Applied Biology 92, 71–80

[CIT0041] Reinstadler A Muller J Czembor JH Piffanelli P Panstruga R 2010 Novel induced *mlo* mutant alleles in combination with site-directed mutagenesis reveal functionally important domains in the heptahelical barley *Mlo* protein. BMC Plant Biology 10, 31. 10.1186/1471-2229-10-31 PMC2844067 20170486

[CIT0042] Roos J Hopkins R Kvarnheden A Dixelius C 2011 The impact of global warming on plant diseases and insect vectors in Sweden. European Journal of Plant Pathology 129, 9–19

[CIT0043] Salamati S Reitan L 2006 *Ramularia collo-cygni* on spring barley, an overview of its biology and epidemiology. In: Proceedings of the 1st European Ramularia Workshop March 2006. Göttingen, Germany: Georg-August-Universität pp 19–35

[CIT0044] Saville RJ Gosman N Burt CJ Makepeace J Steed A Corbitt M Chandler E Brown JKM Boulton MI Nicholson P 2012 The ‘Green Revolution’ dwarfing genes play a role in disease resistance in *Triticum aestivum* and *Hordeum vulgare* . Journal of Experimental Botany 63, 1271–1283 22090435 10.1093/jxb/err350 PMC3276090

[CIT0045] Schützendübel A Stadler M Wallner D von Tiedemann A 2008 A hypothesis on physiological alterations during plant ontogenesis governing susceptibility of winter barley to *Ramularia* leaf spot. Plant Pathology 57, 518–526

[CIT0046] Schulz B Boyle C 2005 The endophytic continuum. Mycological Research 109, 661–686 16080390 10.1017/s095375620500273x

[CIT0047] Shaner G Finney RE 1977 Effect of nitrogen-fertilization on expression of slow-mildewing resistance in knox wheat. Phytopathology 67, 1051–1056

[CIT0048] St Clair DA 2010 Quantitative disease resistance and quantitative resistance loci in breeding. Annual Review of Phytopathology 48, 247–268 10.1146/annurev-phyto-080508-081904 19400646

[CIT0049] Summers RW Brown JKM 2013 Constraints on breeding for disease resistance in commercially competitive wheat cultivars. Plant Pathology in press (10.1111/ppa.12165).

[CIT0050] Taylor JMG Paterson LJ Havis ND 2010 A quantitative real-time PCR assay for the detection of *Ramularia collo-cygni* from barley (*Hordeum vulgare*). Letters in Applied Microbiology 50, 493–499 20337932 10.1111/j.1472-765X.2010.02826.x

[CIT0051] Thomas WTB Baird E Fuller JD Lawrence P Young GR Russell J Ramsay L Waugh R Powell W 1998 Identification of a QTL decreasing yield in barley linked to *Mlo* powdery mildew resistance. Molecular Breeding 4, 381–393

[CIT0052] Tottman DR Makepeace RJ 1979 Explanation of the decimal code for the growth-stages of cereals, with illustrations. Annals of Applied Biology 93, 221–234

[CIT0053] van Ooijen JW 2004 MapQTL 5, software for the mapping of quantitative trait loci in experimental populations. Wageningen, The Netherlands: Kyazma

[CIT0054] van Ooijen JW Voorrips RE 2001 JoinMap 3.0 software for the calculation of genetic linkage maps. Wageningen, The Netherlands: Plant Research International

[CIT0055] Walters DR Avrova A Bingham IJ 2012 Control of foliar diseases in barley: towards an integrated approach. European Journal of Plant Pathology 133, 33–73

[CIT0056] Walters DR Havis ND Oxley SJP 2008 *Ramularia collo-cygni*: the biology of an emerging pathogen of barley. Fems Microbiology Letters 279, 1–7 18070071 10.1111/j.1574-6968.2007.00986.x

[CIT0057] West JS Townsend JA Stevens M Fitt BDL 2012 Comparative biology of different plant pathogens to estimate effects of climate change on crop diseases in Europe. European Journal of Plant Pathology 133, 315–331

[CIT0058] Wolter M Hollricher K Salamin F Schulze-Lefert P 1993 The *mlo* resistance alleles to powdery mildew infection in barley trigger a developmentally controlled defense mimic phenotype. Molecular and General Genetics 239, 122–128 8510641 10.1007/BF00281610

