# Supplementary material for: A trade off between mlo resistance to powdery mildew and increased susceptibility of barley to a newly important disease, Ramularia leaf spot
Source: J Exp Bot. 2014 Jan 7;65(4):1025–37. doi: 10.1093/jxb/ert452 (PMC3935564; doi:10.1093/jxb/ert452)
Supplement: Supplementary Data [file supp_65_4_1025__index.html]

A trade off between mlo resistance to powdery mildew and increased susceptibility of barley to a newly important disease, Ramularia leaf spot — A trade off between mlo resistance to powdery mildew and increased susceptibility of barley to a newly important disease, Ramularia leaf spot — A trade off between mlo resistance to powdery mildew and increased susceptibility of barley to a newly important disease, Ramularia leaf spot — Supplementary Data 

# A trade off between *mlo* resistance to powdery mildew and increased susceptibility of barley to a newly important disease, Ramularia leaf spot

## Supplementary Data

Data files

**Files in this Data Supplement:**

- Supplementary Data - Supplementary Data
