# Supplementary material for: A trade off between mlo resistance to powdery mildew and increased susceptibility of barley to a newly important disease, Ramularia leaf spot
Source: J Exp Bot. 2014 Jan 7;65(4):1025–37. doi: 10.1093/jxb/ert452 (PMC3935564; doi:10.1093/jxb/ert452)
Supplement: Supplementary Data [file supp_ert452_jexbot110254_file001.pdf]

G.R.D. McGrann, A. Stavrinides, J. Russell, M.M. Corbitt, A. Booth, L. Chartrain, W.T.B. Thomas & J.K.M. Brown (2014)

A trade-off between *mlo* resistance to powdery mildew and increased susceptibility of barley to a newly important disease, Ramularia leaf spot. *Journal of Experimental Botany*.

Supplementary material

Figure S1. Genetic linkage map of 122 single-nucleotide polymorphism (SNP) markers and the MLO gene in 196 doubled-haploid progeny of the spring barley cultivars Power x Braemar.

Figure S2. Genetic linkage map of 120 single-nucleotide polymorphism (SNP) markers in 144 doubled-haploid progeny of the spring barley cultivars Decanter x Cocktail.

McGrann *et al.* (2014) Figure S2: SNP-based genetic map of Power x Braemar

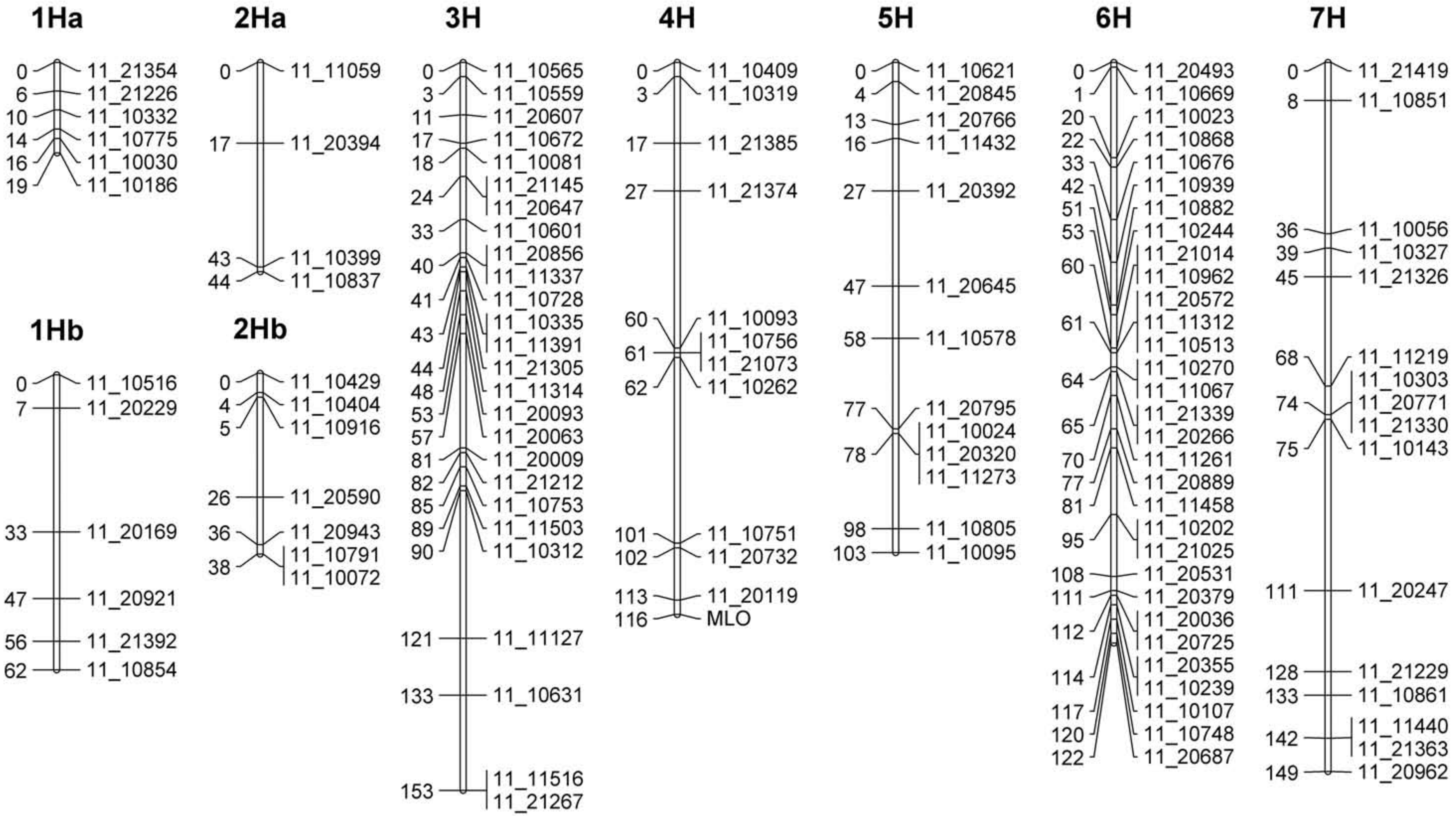

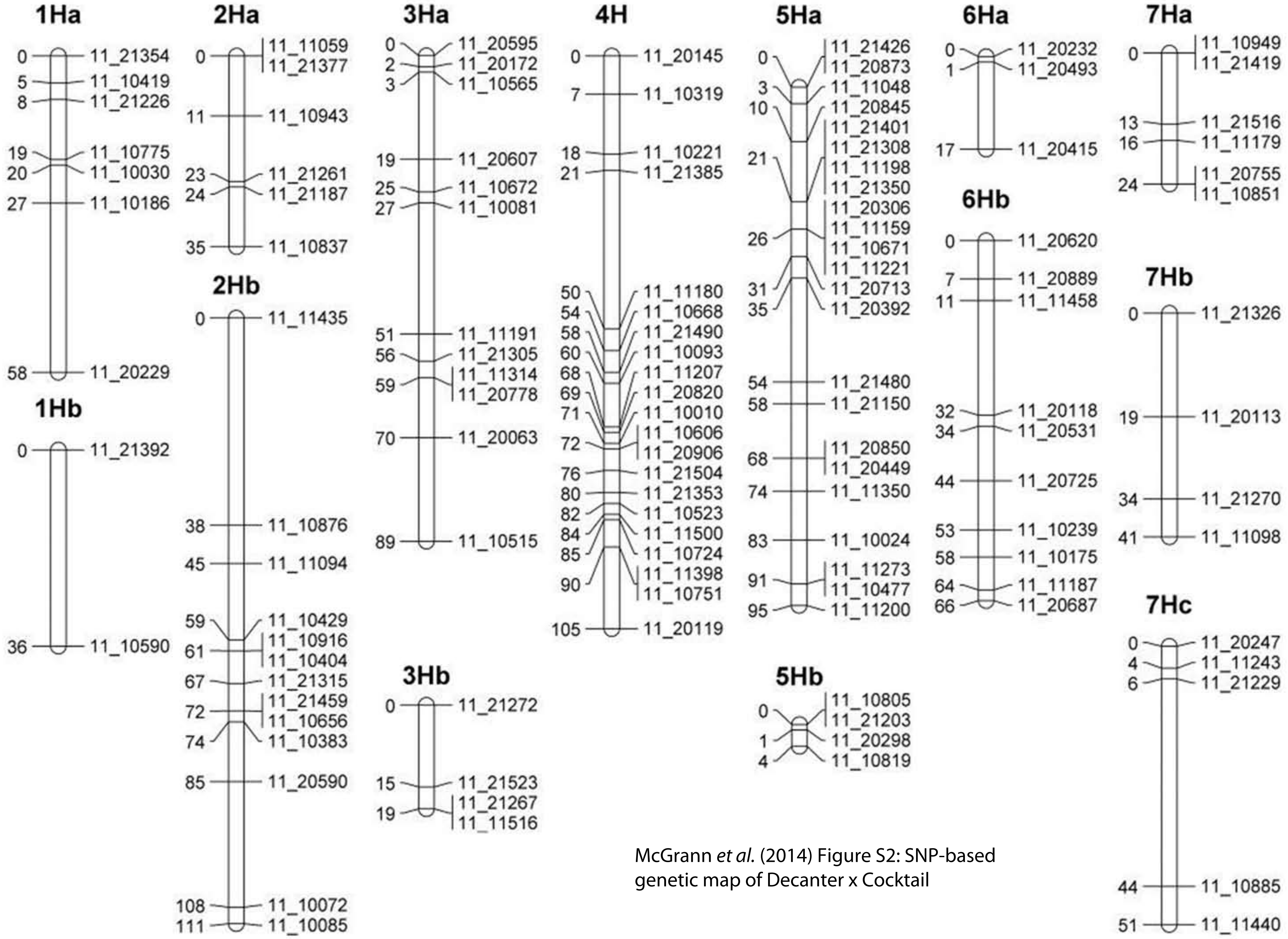

McGrann *et al.* (2014) Figure S2: SNP-based genetic map of Decanter x Cocktail
